# Synthesis and pharmacological characterization of potent, selective, and orally bioavailable isoindoline class dipeptidyl peptidase IV inhibitors

**DOI:** 10.1186/2191-2858-1-7

**Published:** 2011-09-12

**Authors:** Noriyasu Kato, Mitsuru Oka, Takayo Murase, Masahiro Yoshida, Masao Sakairi, Mirensha Yakufu, Satoko Yamashita, Yoshika Yasuda, Aya Yoshikawa, Yuji Hayashi, Masahiro Shirai, Yukie Mizuno, Mitsuaki Takeuchi, Mitsuhiro Makino, Motohiro Takeda, Takuji Kakigami

**Affiliations:** 1 Central Research Laboratory, Sanwa Kagaku Kenkyusho, Co., Ltd., 363 Shiosaki, Hokusei-cho, Inabe-city, Mie 511-0406, Japan; 2 Sanwa Kagaku Kenkyusho, Co., Ltd., 35 Higashisotobori-cho, Higashi-ku, Nagoya 461-8631, Japan; 3 Xinjiang Medical University, Urumqi 830011, China

## Abstract

Focused structure-activity relationships of isoindoline class DPP-IV inhibitors have led to the discovery of **4b **as a highly selective, potent inhibitor of DPP-IV. *In vivo *studies in Wistar/ST rats showed that **4b **was converted into the strongly active metabolite **4l **in high yield, resulting in good *in vivo *efficacy for antihyperglycemic activity.

## 1. Background

**Figure 1:**
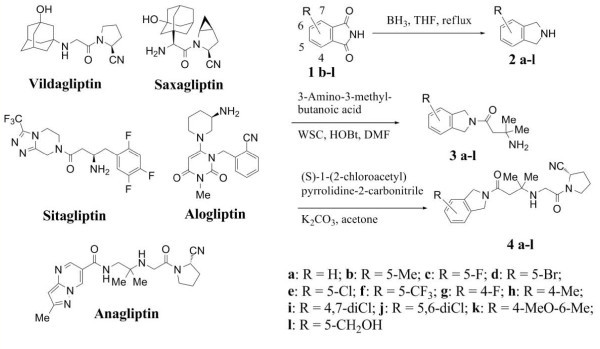
**Some gliptins and isoindoline class DPP-4 inhibitors**.

With the advent of sitagliptin (MK-0431) and vildagliptin (LAF-237), doubt no longer exists regarding the potential of dipeptidyl peptidase IV (DPP-IV; CD26; E.C. 3.4.14.5) inhibitors for the treatment of type 2 diabetes [1-4]. Hence, intensive research efforts are being continued, and have led to the discovery of a number of potent DPP-IV inhibitors (Figure 1) [5-9]. Research on second-generation DPP-IV inhibitors has focused on selectivity for DPP-IV over other proline-specific dipeptidyl peptidases, especially DPP8/9, since it has been suggested that inhibition of DDP-8/9 is associated with severe toxicity [10,11]. In addition, the results of recent clinical trials have indicated that prolonged and marked inhibition of DPP-IV would be beneficial for severely diabetic patients [12,13]. The requirement for prolonged, high exposure in humans imposes stringent requirements on the safety profiles and ADME properties of back-up compounds. In this article, we describe our preliminary results with potent and selective isoindoline class DPP-IV inhibitors with respect to CYP, cytochrome P450, induction, and rodent PK, studies as well as inhibition of DPP-IV activity.

## 2. Results and discussion

Very recently, Jiaang and co-workers reported that prolinenitrile-based inhibitors with heterocyclic rings showed high selectivity and potency for DPP-IV as well as *in vivo *efficacy compared to vildagliptin [14]. We had also pursued the possibility of isoindoline class DDP-IV inhibitors and found their high potency and excellent *in vivo *efficacy [15]. Thus, isoindolines were synthesized as shown in Figure 1 and evaluated *in vitro *for their ability to inhibit human recombinant DPP-IV and were also screened for their selectivity over DPP-8/9 by a fluorescence assay using glycyl-proline 7-amino-4-methylcoumarin (H-Gly-Pro-AMC). The inhibitory potency is reported as the IC_50 _value (Table 1). All the compounds had excellent selectivity for DPP-IV over the other related peptidases. Monosubstitution at positions around the benzene ring of **4a **was well tolerated, while retaining a high level of selectivity. Disubstitution, however, led to a slight decrease in potency (**4j**, **k**). Disappointingly, most compounds showed CYP induction of either or both of the two enzymes. Eventually, **4b **was subjected to further investigation.

**Table 1:** Inhibition of DPP-IV, -8 and -9 activity by 1,3-dihydroisoindoline derivatives 4, their metabolic clearance by rat and human and their enzyme-inducing (CYP1A, CYP2B, and CYP3A) capacity

Compound 4	R	IC_50 _(nM)			CL'int (L/h/Kg)		Enzyme induction (rat)		
		
		DPP-IV	DPP8	DPP9	Rat	Human	CYP1A	CYP2B	CYP3A
**a**	-H	2.3	> 100,000	> 100,000	1.3	0.2	+	+	+
**b**	5-Me	3.4 (28)^c^	59,000	> 100,000	2.3	0.1	-	-	-
**c**	5-F	1.9	> 100,000	> 100,000	2.6	4.8	-	+	N.T.^b^
**d**	5-Br	3.0	36,000	> 100,000	N.T.^b^	N.T.^b^	-	+	N.T.^b^
**e**	5-Cl	4.8	44,000	> 100,000	N.T.^b^	N.T.^b^	-	+	N.T.^b^
**f**	5-CF_3_	5.4	> 100,000	> 100,000	N.T.^b^	N.T.^b^	+	+	N.T.^b^
**g**	4-F	2.6	> 100,000	> 100,000	N.T.^b^	N.T.^b^	-	+	N.T.^b^
**h**	4-Me	4.0	> 100,000	> 100,000	N.T.^b^	N.T.^b^	-	+	N.T.^b^
**i**	4,7-diCl	2.6	> 100,000	> 100,000	N.T.^b^	N.T.^b^	+	-	N.T.^b^
**j**	5,6-diCl	22	> 100,000	74,000	N.T.^b^	N.T.^b^	+	+	N.T.^b^
**k**	4-MeO-6-Me	16	> 100,000	> 100,000	N.T.^b^	N.T.^b^	-	+	N.T.^b^
**l**	5-CH_2_OH	1.9^a^	N.T.^b^	N.T.^b^	N.T.^b^	N.T.^b^	-	-	N.T.^b^

^a^IC_50 _determined with respect to human plasma DPP-IV in separate experiments.

^b^Not tested.

*In vivo *PK studies on **4b **showed a short plasma half-life and reduced AUC when dosed intravenously (Table 2). Apparently, the reduction in AUC was partly due to a very high clearance. On the other hand, oral administration of **4b **showed an improved half-life and a dose-dependent increase in AUC. As it was estimated from the PK profiles that 3-10 mg/kg doses of **4b **would provide > 50% inhibition of DPP-IV for several hours, we tried to briefly examine the potency of **4b **in oral glucose tolerance tests (OGTT).

**Table 2:** PK parameters of 4b in SD rats

Route	Dose (mg/kg)	***t***_**1/2α**_ (h)	***t***_**1/2α**_ (h)	**Vd**_**ss**_ (L/kg)	**CL**_**p**_ (L/h/kg)	**C**_**max**_ (ng/mL)	***T***_**max**_ (h)	**AUC**_**0-9 h**_ (ng h/mL)	BA (%)
Iv	1	0.062	0.27	8.28	26.2	ND	ND	39	
po	3	ND	1.37	ND	ND	37	0.25	33	27.7
	10	ND	1.50	ND	ND	229	0.25	156	39.9

**Figure 2:**
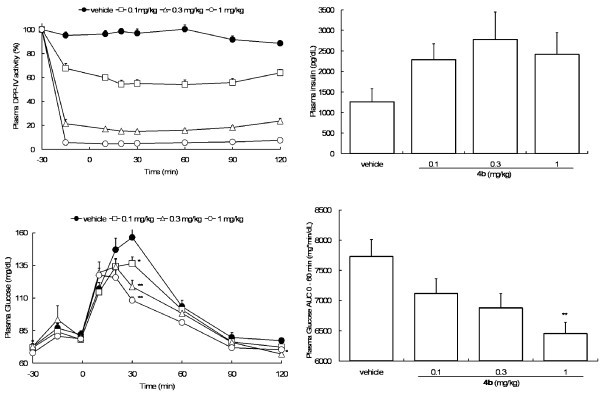
**Pharmacological data on 4b**. Top left, plasma DPP-IV activity (% change from -30 min value) in Wistar/ST rats. Data are given as mean ± SEM (*n *= 7). Top right, plasma insulin (pg/dL) at 10 min after glucose challenge in Wistar/ST rats. Data are given as mean ± SEM (*n *= 7). Bottom left, blood glucose during OGTT in Wistar/ST rats. Data are given as mean ± SEM (*n *= 7). Asterisks indicate significance from vehicle control at *p *< 0.05 (*) and *p *< 0.01 (**) by Dunnett's test. Bottom right, blood glucose AUC (mg min/dL) determined between 0 and 60 min during OGTT in Wistar/ST rats. Data are given as mean ± SEM (*n *= 7). **Significant difference from vehicle control by Dunnett's test (*p *< 0.01).

Fasted male Wistar/ST rats received either vehicle or **4b **at different oral doses (Figure 2). After 30 min (*t *= 0), oral glucose challenges (1 g/kg) were conducted and then plasma DPP-IV activities and blood glucose levels were monitored at various intervals over a 2 h period. Selected data are shown in Figure 2. To our surprise, the 1 mg/kg dose of **4b **resulted in 95% inhibition of plasma DPP-IV activity within 30 min post-dose and inhibition of greater than 90% was maintained throughout the study. The inhibitory effect was dose-dependent, and even the 0.1 mg/kg dose produced 30% inhibition. Similarly, reduction of glucose levels paralleled DPP-IV inhibition and a reduction of 18% was observed at a dose of 1 mg/kg. In addition, increased insulin levels at 10 min post-challenge strongly suggested preservation of active GLP-1.

The high clearance of **4b **suggested that the unexpected *in vivo *efficacy might be explained by the presence of active metabolites. Therefore, further PK studies were conducted. A major metabolite was detected by LC-MS analysis and its structure was determined by comparison of the LC retention time and MS/MS fragmentation pattern with synthetic standards. Consequently, **4l **was identified as a very active metabolite, which showed a reasonable degree of systemic exposure by virtue of *in vivo *conversion as high as 60%.

## 3. Conclusions

In summary, the focused, small SARs of the isoindoline derivatives have led to the discovery of **4b **as a highly selective, potent inhibitor of DPP-IV. The *in vivo *studies showed that the active metabolite **4l **had a very high inhibitory potency with respect to DPP-IV. Consequently, we abandoned further development of compounds in this series. However, on the basis of the results described here, we found anagliptin, which has advanced into PIII trials, to have improved safety profiles and PK parameters. This article is also intended to provide information on the scope and limitations of isoindoline-based DPP-IV inhibitors and to facilitate research on the new generation DPP-IV inhibitors.

## 4. Methods

### 4.1. Compound synthesis

#### 4.1.1. General

All commercially available reagents and solvents were used as-received. All reactions were carried out using oven-dried flasks or glassware, and mixtures were stirred with stirring bars and concentrated using a standard rotary evaporator unless otherwise noted. Procedures for preparation of all intermediates **2 **and **3 **were described previously [15]. The ^1^H NMR spectra were recorded by a JEOL JNM-ECP400 spectrometer operating at 400 MHz in DMSO-d_6 _at 25°C with tetramethylsilane as the internal standard. The data are reported as follows: chemical shift in ppm (δ), integration, multiplicity (s = singlet, d = doublet, t = triplet, q = quartet, br = broad singlet, m = multiplet), and coupling constant (Hz). LC/MS spectra were determined on a Waters ZMD2000 equipped with a Waters 2690 injector and a PDA detector operating at 210-400 nm and interfaced with a Micromass ZMD mass spectrometer.

#### 4.1.2. Representative procedure for preparation of pyrrolidine carbonitrile 4; (S)-1-(2-(4-(Isoindolin-2-yl)-2-methyl-4-oxobutan-2-ylamino)acetyl)pyrrolidine-2-carbonitrile HCl Salt (4a)

A solution of (*S*)-1-(2-chloroacetyl)pyrrolidine-2-carbonitrile (467 mg, 2.70 mmol) in acetone (5.0 mL) was added drop-by-drop to an ice-cooled stirred suspension of **3a **(550 mg, 2.50 mmol), K_2_CO_3 _(370 mg, 2.70 mmol), and NaI (200 mg, 1.30 mmol) in acetone (20 mL). The reaction mixture was stirred at room temperature overnight. The resulting mixture was filtered to remove insoluble materials, and concentrated under reduced pressure. The residue was purified by column chromatography on silica gel (CH_2_Cl_2_/MeOH = 20/1) to give 540 mg (61%) of **4a **of the free base. To an ice-cooled solution of **4a **of the free base (250 mg, 0.70 mmol), 1,4-dioxane (5.0 mL) was added 4N-HCl/1,4-dioxane (180 μL, 0.72 mmol). The reaction mixture was stirred at 0°C for 1 h and then evaporated to yield the title compound (240 mg, Y. 88%). ^1^H NMR 1.38 (6H, s), 2.00-2.22 (4H, m), 2.85-2.90 (2H, m), 3.30-4.10 (4H, m), 4.69 (2H, s), 4.87 (2H, s), 4.80-4.85 (1H, m), 7.25-7.40 (4H, m); MS *m/z *355 (M+H)^+^.

#### 4.1.3. (S)-1-(2-(2-Methyl-4-(5-methylisoindolin-2-yl)-4-oxobutan-2-ylamino)acetyl)pyrrolidine-2-carbonitrile HCl Salt (4b)

Colorless solid (92%). ^1^H NMR 1.41 (6H, s), 1.99-2.11(2H, m), 2.18-2.24 (2H, m), 2,32 (3H, s), 2.88-2.98 (2H, m), 3.21-3.39 (2H, m), 3.50-3.57 (1H, m), 3.68-3.72 (1H, m), 4.06-4.10 (1H, m), 4.66 (2H, s), 4.86 (2H, sm), 7.13-7.28 (3H, m), 9.29 (2H, brs); MS *m/z *369 (M+H)^+^.

#### 4.1.4. (S)-1-(2-(4-(5-Fluoroisoindolin-2-yl)-2-methyl-4-oxobutan-2-ylamino)acetyl)pyrrolidine-2-carbonitrile HCl Salt (4c)

Colorless solid (31%). ^1^H NMR 1.40 (6H, s), 2.02-2.08(2H, m), 2.19-2.22 (2H, m), 2.88-2.89 (2H, m), 3.50-3.69 (2H, m), 4.04-4.07 (2H, m), 4.67-4.70 (2H, m), 4.85-4.89 (3H, m), 7.16 (1H, t, *J *= 9.2 Hz), 7.24 (1H, t, *J *= 9.2 Hz), 7.37-7.44 (1H, m), 9.10 (2H, brs); MS *m/z *373 (M+H)^+^.

#### 4.1.5. (S)-1-(2-(4-(5-Bromoisoindolin-2-yl)-2-methyl-4-oxobutan-2-ylamino)acetyl)pyrrolidine-2-carbonitrile HCl Salt (4d)

Colorless solid (81%). ^1^H NMR 1.35 (6H, s), 1.95-2.05 (2H, m), 2.12-2.18 (2H, m), 2.83 (2H, s) 3.70-4.05 (4H, m), 4.62-4.72 (2H, m), 4.78-4.84 (3H, m), 7.29-7.60 (3H, m), 8.21 (2H, brs); MS *m/z *423 (M+H)^+^.

#### 4.1.6. (S)-1-(2-(4-(5-Chloroisoindolin-2-yl)-2-methyl-4-oxobutan-2-ylamino)acetyl)pyrrolidine-2-carbonitrile HCl salt (4e)

Colorless solid (38%). ^1^H NMR 1.65 (6H, s), 2.20-2.35 (4H, m), 2.90-3.35 (2H, m), 3.70-4.40 (4H, m), 4.75-5.00 (5H, m), 7.20-7.30 (3H, m); MS *m/z *389 (M+H)^+^.

#### 4.1.7. (S)-1-(2-(2-Methyl-4-oxo-4-(5-(trifluoromethyl)isoindolin-2-yl)butan-2-ylamino)acetyl)pyrrolidine-2-carbonitrile HCl salt (4f)

Colorless solid (37%). ^1^H NMR 1.41 (6H, s), 1.98-2.09 (2H, m), 2.18-2.25 (2H, m), 2.92 (2H, d, *J *= 3.3 Hz), 3.50-3.54 (1H, m), 3.67-3.72 (1H, m), 4.00-4.13 (2H, m), 4.78 (2H, s), 4.87 (1H, dd, *J *= 3.3 and 7.3 Hz), 4.97 (2H, s), 7.59-7.64 (1H, m), 7.69 (1H, d, *J *= 8.1 Hz), 7.76-7.80 (1H, m), 9.19 (2H, brs); MS *m/z *423 (M+H)^+^.

#### 4.1.8. (S)-1-(2-(4-(4-Fluoroisoindolin-2-yl)-2-methyl-4-oxobutan-2-ylamino)acetyl)pyrrolidine-2-carbonitrile HCl salt (4g)

Colorless solid (23%). ^1^H NMR 1.40 (6H, s), 2.01-2.09(2H, m), 2.18-2.25 (2H, m), 2.92-2.94 (2H, m), 3.51-3.53 (1H, m), 3.66-3.72 (1H, m), 4.00-4.13 (2H, m), 4.75 (2H, s), 4.85-4.87 (1H, m), 4.97 (1H, s), 7.16 (1H, t, *J *= 8.8 Hz), 7.21-7.25 (1H, m), 7.37-7.43 (1H, m), 9.18 (2H, brs); MS *m/z *373 (M+H)^+^.

#### 4.1.9. (S)-1-(2-(2-Methyl-4-(4-methylisoindolin-2-yl)-4-oxobutan-2-ylamino)acetyl)pyrrolidine-2-carbonitrile HCl salt (4h)

Colorless solid (33%). ^1^H NMR 1.41 (6H, s), 2.03-2.11(2H, m), 2.17-2.24 (2H, m), 2.26 (3H, s), 2.95 (2H, d, *J *= 8.9 Hz), (2H, m), 3.40-3.66 (2H, m), 3.97-4.10 (2H, m), 4.25-4.31 (1H, m), 4.68 (2H, s), 4.88 (2H, s), 7.11-7.25 (3H, m), 9.29 (2H, brs); MS *m/z *369 (M+H)^+^.

#### 4.1.10. (S)-1-(2-(4-(4,7-Dichloroisoindolin-2-yl)-2-methyl-4-oxobutan-2-ylamino)acetyl)pyrrolidine-2-carbonitrile HCl salt (4i)

Colorless solid (69%). ^1^H NMR 1.41 (6H, s), 2.03-2.10 (2H, m), 2.19-2.25 (2H, m), 2.94-2.97 (2H, m), 3.67-3.72 (2H, m), 4.03-4.14 (2H, m), 4.77 (2H, s), 4.86(1H, dd, *J *= 4.4 and 7.3 Hz), 5.00 (2H, s), 7.48 (2H, s), 9.18 (2H, brs); MS *m/z *423 (M+H)^+^.

#### 4.1.11. (S)-1-(2-(4-(5,6-Dichloroisoindolin-2-yl)-2-methyl-4-oxobutan-2-ylamino)acetyl)pyrrolidine-2-carbonitrile HCl salt (4j)

Colorless solid (10%). ^1^H NMR 1.65 (6H, s), 2.20-2.35 (4H, m), 2.90-3.35 (2H, m), 3.70-4.40 (4H, m), 4.80-4.95 (5H, m), 7.37-7.44 (2H, m); MS *m/z *423 (M+H)^+^.

#### 4.1.12. (S)-1-(2-(4-(4-Methoxy-6-methylisoindolin-2-yl)-2-methyl-4-oxobutan-2-ylamino)acetyl)pyrrolidine-2-carbonitrile HCl salt (4k)

Colorless solid (77%). ^1^H NMR 1.55-1.70 (6H, m), 2.20-2.35 (4H, m), 2.37 (3H, s), 2.80-3.40 (2H, m), 3.60-4.45 (7H, m), 4.65-4.90 (5H, m), 6.55-6.75 (2H, m); MS *m/z *399 (M+H)^+^.

#### 4.1.13. (S)-1-(2-(4-(5-(Hydroxymethyl)isoindolin-2-yl)-2-methyl-4-oxobutan-2-ylamino)acetyl)pyrrolidine-2-carbonitrile HCl salt (4l)

Colorless solid (81%). ^1^H NMR 1.41 (6H, s), 2.0-2.25 (4H, m), 2.92 (2H, m), 3.5-4.1 (4H, m), 4.5-4.9 (7H, m), 7.2-7.4 (3H, m), 9.28 (2H, brs); MS *m/z *385 (M+H)^+^.

### 4.2. Biological evaluation

#### 4.2.1. In vitro assay for DPP-IV inhibition

Inhibition of DPP-IV activity was determined by measuring the rate of hydrolysis of a surrogate substrate, H-Gly-Pro-7-amino-4-methylcoumarin (H-Gly-Pro-AMC). Human recombinant DPP-IV was purchased from R&D Systems, Minneapolis, MN. 10 μL of appropriately diluted solutions of the test compounds in water was added to 96-well microtiter plates, followed by the addition of 40 μL of DPP-IV diluted in assay buffer (25 mM HEPES, 140 mM NaC1, 0.1 mg/mL BSA, pH 7.8). After a 30-min preincubation at room temperature, the reaction was initiated by the addition of 50 μL of the assay buffer containing 0.2 mM H-Gly-Pro-AMC. After incubation at room temperature for 20 min, the reaction was stopped by the addition of 100 μL of 25% aqueous acetic acid and fluorescence was measured using an excitation wavelength of 390 nm and an emission wavelength of 460 nm. A standard curve for AMC was generated by adding 0.2-20 μmol of AMC to buffer solutions containing 12.5% aqueous acetic acid. The inhibitory rate relative to the control without inhibitor was calculated and IC_50 _values were determined by nonlinear regression (GraphPad Prism 4, ver. 4.03 software).

#### 4.2.2 In vitro assays for inhibition of DPP-8 and DPP-9

Human DPP-8 and DPP-9 were expressed in baculovirus-infected Sf9 insect cells and purified using *His*-tagged protein purification resins. Inhibition of DPP-8 and -9 activities was determined as described above. 10 μL of appropriately diluted aqueous solutions of the test compounds was added to 96-well microtiter plates, followed by the addition of 50 μL of 1.0 mM H-Gly-Pro-AMC in buffer solution (50 mM HEPES, 0.1 mg/mL BSA, pH 8.0). The reaction was initiated by the addition of 40 μL of the enzyme solution diluted in the assay buffer. After incubation at room temperature for 30 min, the reaction was stopped by the addition of 100 μL of 25% aqueous acetic acid and fluorescence was measured using an excitation wavelength of 390 nm and an emission wavelength of 460 nm.

#### 4.2.3. In vivo assay methods

All procedures were approved by the Sanwa Kagaku Kenkyusho Institutional Animal Care and Use Committee. 7-week old Wistar/ST rats were housed under standard conditions and allowed free access to water and a commercial diet for at least 5 days. The rats were fasted overnight prior to dosing and then received **4b **orally at doses of 0.1-1 mg/kg or vehicle as a 5 mL/kg aqueous solution 30 min before glucose challenge. After an oral glucose challenge (5 mL/kg of an aqueous solution of 20% glucose), blood samples were collected from the tail vein of each animal into heparin-containing tubes at serial time points for 2 h. Plasma was prepared immediately, frozen, and stored at -20°C prior to analysis.

#### 4.2.4 Inhibition of rat plasma DPP-IV ex vivo

Plasma DPP-IV activity was determined as described above. A 20 μL plasma sample was mixed with 5 μL of reaction buffer (140 mM NaCl, and 10 mM KCl, 25 mM Tris-HCl, pH 7.4, 1% bovine serum albumin) and 10 μL of buffer containing 60 μM H-Gly-Pro-AMC. After incubation at room temperature for 30 min, the reaction was stopped by the addition of 20 μL of 25% aqueous acetic acid and fluorescence was measured using an excitation wavelength of 360 nm and an emission wavelength of 460 nm.

#### 4.2.5 Measurement of plasma glucose and insulin concentrations

Plasma glucose and insulin were determined with a glucometer (Glutest Pro; SKK, Japan) and a rat insulin ELISA kit (Shibayagi, Japan), respectively, according to the manufacturer's instructions. Statistical analyses were performed using Microsoft Excel. Individual comparisons among more than two experimental groups were assessed using ANOVA, with Fisher's Least Significant Difference *post hoc *test. Differences were considered significant at *P *values < 0.05. Analysis of dose-response data was performed by Dunnett's test.

#### 4.2.6 Pharmacokinetics (PK) in rats

Sprague-Dawley (SD) rats were housed under standard conditions and allowed free access to water and a commercial diet. On the day before the experiment, rats were fasted overnight and for the first 12 h of the experiment. Compounds **4b **were prepared in a saline/ethanol vehicle (50/50 v/v) at appropriate concentrations of **4b **as an intravenous (iv) injection of 1 mL/kg via the femoral vein and as a suspension in 5% gum arabic solution for oral (po) administration. Blood samples were collected from the jugular vein of each animal with a heparinized syringe under diethyl ether anesthesia at serial time points for 24 h after drug administration. Plasma was obtained by centrifugation at 4°C and stored at -70°C until analysis. Protein precipitation was carried out by the addition of the internal standard solution (70% CH_3_CN with 0.2% acetic acid) to samples. The tubes underwent vigorous shaking and centrifugation for 5 min; then the supernatant was subjected to LC/MS/MS analysis. Peak areas were determined using Xcalibur^® ^software (Thermo Electron Corporation, UK) and AUC values were calculated by the trapezoidal rule.

### 4.3. Metabolic stability

The incubation mixture containing 0.25 mg of rat or human liver microsomes was preincubated with an NADPH-generating system for 5 min at 37°C. The reaction was started by the addition of 5 μL of a DMSO solution containing the test compound (5 μM). At *t *= 0 and at two additional time points between 0 and 30 min, aliquots (100 μL) were removed and added to termination mixtures (CH_3_CN). Proteins were sedimented by centrifugation and an aliquot of the supernatant was analyzed by LC/MS/MS.

In determinations of the *in vitrot*_1/2_, the analyte/ISTD peak area ratio was converted to percentage of drug remaining by assigning a value of 100% to the peak area ratio at *t *= 0. The slope of the regression line fitted to the log (percentage remaining) versus incubation time relationship (-*k*) was used in the conversion of raw data to the *in vitrot*_1/2 _value. *In vitro *CL_int _was calculated using the following formula.

$$\text{C}{\text{L}}_{\text{int}}=\frac{\text{0}\text{.693}}{\text{In}\;\text{vitro }{\text{t}}_{\text{1/2}}}\times \frac{\text{mL incubation}}{\text{mg microsomes}}\times \frac{\text{45 mg microsomes}}{\text{mg liver}}\times \frac{\text{20 mg liver}}{\text{kg (b}\text{.w}\text{.)}}$$

Enzyme induction was evaluated as follows: Hepatocytes isolated from male SD rats were maintained in culture for 1 day before treatment with the test compound or P-450 inducers. The cells were treated with the test compound (1, 10, 50 μM), β-naphthoflavone (10 μM, CYP1A inducer), phenobarbital (50 μM, CYP2B inducer), dexamethasone (10 μM, CYP3A inducer), or vehicle (0.1% DMSO final volume; used as negative control) for 2 days.

Induction of CYP1A, CYP2B, and CYP3A enzymes was determined based on measurements of 7-ethoxyresorufin O-dealkylation, 7-pentoxyresorufin O-dealkylation, and testosterone 6β-hydroxylation, respectively. Assays were started by the addition of Krebs-Henseleit buffer containing 8 μM 7-ethoxyresorufin, 10 μM 7-pentoxyresorufin, or 250 μM testosterone at a volume of 100 μL per well. After incubation at 37°C for 30 min, aliquots were removed and analyzed by fluorometry (an excitation wavelength of 538 nm and an emission wavelength of 590 nm) or LC/MS/MS to determine the quantities of metabolites formed. Any test compound causing a dose-dependent change equal to or greater than 10% of the positive control (see formula below) was considered an enzyme inducer.

$$\text{\% positive control}=\frac{\text{(activity of test - compound treated cells - activity of negative control)}}{\text{(activity of positive control - activity of negative control)}}\times 100$$

## Competing interests

The authors declare that they have no competing interests.
